# Gender differences in severity of sickle cell diseases in non-smokers

**DOI:** 10.12669/pjms.294.3697

**Published:** 2013

**Authors:** Mehmet Rami Helvaci, Orhan Ayyildiz, Mehmet Gundogdu

**Affiliations:** 1 Mehmet Rami Helvaci, MD, Assoc. Prof. of Internal Medicine, Medical Faculty of the Mustafa Kemal University, Antakya, Turkey.; 2 Orhan Ayyildiz, MD, Professor of Internal Medicine, Faculty of the Dicle University, Diyarbakir, Turkey.; 3 Mehmet Gundogdu, MD, Professor of Internal Medicine, Medical Faculty of the Ataturk University, Erzurum, Turkey.

**Keywords:** Atherosclerosis, Gender differences, Sickle cell diseases, Non-smokers

## Abstract

***Objective:*** To find out gender differences in severity of sickle cell diseases (SCDs) in non-smokers.

***Methods:*** Three groups of SCDs patients on the basis of red blood cell (RBC) transfusions were included. Less than 10 units in their lives were kept in Group-1, Ten units of higher in Group-2 and 50 units or higher as the Third Group. Patients with a history of using one pack of cigarettes -year or above were excluded.

***Results:*** The study included 269 patients. Mean ages of the groups were similar (28.4, 28.5, and 28.9 years, respectively). Prevalences of cases without any RBC transfusion in their lives were 7.2% and 3.7% in females and males, respectively (*p*<0.05). Prevalences of cases without any painful crisis were 13.8% and 6.0% in females and males, respectively (*p*<0.001). There was progressive increase according to mean painful crises, clubbing, chronic obstructive pulmonary disease (COPD), leg ulcers, stroke, chronic renal disease (CRD), pulmonary hypertension, and male ratio from the first towards the third groups (*p*<0.05, nearly for all). Mean ages of mortal cases were 29.1 and 26.2 years in females and males, respectively (*p*>0.05).

***Conclusion:*** The higher painful crises per year, digital clubbing, COPD, leg ulcers, stroke, CRD, pulmonary hypertension, and male ratio of the third group, lower male ratio of patients without any RBC transfusion, lower male ratio of patients without any painful crisis, lower mean ages of male SCDs patients with mortality, and longer overall survival of females in the world could not be explained by well known strong atherosclerotic effects of smoking alone, instead it may be explained by the dominant role of male sex in life.

## INTRODUCTION

Systemic atherosclerosis may be the main cause of aging of human being.^1^^,^^2^ It is an irreversible process initiating at birth, and accelerated by many factors. The accelerating factors known today are collected under the heading of metabolic syndrome. Some reversible components of the syndrome are overweight, hypertriglyceridemia, hyperbetalipoproteinemia, dyslipidemia, white coat hypertension, impaired fasting glucose, impaired glucose tolerance, and smoking for the development of terminal consequences such as obesity, diabetes mellitus (DM), hypertension (HT), coronary heart disease (CHD), chronic obstructive pulmonary disease (COPD), cirrhosis, chronic renal disease (CRD), peripherial arterial disease (PAD), stroke, and other end-organ injuries.^3^^-^^6^

Sickle cell diseases (SCDs) are a prototype of the accelerated systemic atherosclerotic process^7^, by which we can observe terminal consequences of the metabolic syndrome very early in life. SCDs are caused by homozygous inheritance of the hemoglobin S (Hb S). As a less polar amino acid, glutamic acid is replaced with valine in the sixth position of beta chain of the Hb S, and it promotes polymerisation of the Hb S. So Hb S causes erythrocytes to change their normal elastic structures to hard bodies. The rigidity of erythrocytes instead of shapes is the central pathology of the SCDs. The sickling process is probably present in whole life, but exaggerated with various stresses.

The erythrocytes can take their normal elastic structures after normalization of the stresses, but after repeated cycles of sickling and unsickling, they become hard bodies, permanently. The rigid cells induced chronic endothelial damage causes tissue ischemia, infarctions, and end-organ failures even in the absence of obvious vascular occlusions due to the damaged and edematous endothelium. We tried to understand whether or not there is a gender difference in clinical severity of SCDs in the absence of well known strong atherosclerotic effects of smoking.

## METHODOLOGY

The study was performed in the Hematology Service of the Mustafa Kemal University between March 2007 and April 2013. All patients with SCDs were enrolled into the study. SCDs are diagnosed by the hemoglobin electrophoresis performed via high performance liquid chromatography (HPLC). Their medical histories including numbers of painful crises per year, units of transfused red blood cell (RBC) in their lives, regular alcohol consumption, smoking habit, leg ulcers, and stroke were recorded. Cases with a history of one pack-year or above were accepted as smokers, and they were excluded from the study.

A check up procedure including serum iron, total iron binding capacity, serum ferritin, serum creatinine value on three occasions, hepatic function tests, markers of hepatitis viruses A, B, and C and human immunodeficiency virus, an electrocardiography, a Doppler echocardiography, a Doppler ultrasonography to evaluate the liver and portal blood flow, and a computed tomography of the brain was performed. Cases with acute painful crisis or another inflammatory event were treated at first, and then the spirometric pulmonary function tests to diagnose COPD, the Doppler echocardiography to measure the systolic blood pressure (BP) of pulmonary artery, renal and hepatic function tests, and measurement of serum ferritin level were performed on the silent phase.

The criterion for diagnosis of COPD is post-bronchodilator forced expiratory volume in onesecond/forced vital capacity of less than 70%.^8^ Systolic BP of the pulmonary artery of 40 mmHg or higher during the silent phase is accepted as pulmonary hypertension.^9^ CRD is diagnosed with a permanently elevated serum creatinine level of 1.3 mg/dL or higher on the silent phase. Cases with renal transplantation were put into the CRD group. Cirrhosis is diagnosed with hepatic function tests, Doppler ultrasonographic findings, and ascites without any histologic procedure in the absence of indication. Digital clubbing is diagnosed by determining the ratio of distal phalangeal diameter to interphalangeal diameter which is required to be higher than 1.0, and with the presence of Swamroth sign.^10^^,^^11^

Associated thalassemias are detected by, serum ferritin, and the hemoglobin electrophoresis. A stress electrocardiography was performed in cases with an abnormal electrocardiography and/or history of angina pectoris. Coronary angiography was advised just for the stress electrocardiography positive cases. So CHD was diagnosed either with the Doppler echocardiographic findings as movement disorders in cardiac walls or angiographically. Eventually, cases with RBC transfusions of less than 10 units in their lives were collected into the first, 10 units or higher into the second, and 50 units or higher into the third groups. Mann-Whitney U test, Independent-Samples t test, and comparison of proportions were used as the methods of statistical analyses.

## RESULTS

The study included 269 patients with SCDs (132 males and 137 females). There were 88 cases (32.7%) in the first, 103 cases (38.2%) in the second, and 78 cases (28.9%) in the third groups without any significant difference in distribution (*p*>0.05 between all). There was not a significant difference according to the prevalence of associated thalassemias between the three groups, either (Table-I). Mean ages of the three groups were similar, too (28.4, 28.5, and 28.9 years, respectively, *p*>0.05 between all). The mean units of transfused RBCs were 4.0, 21.2, and 97.0, respectively (*p*<0.001 between all).

**Table-I:** Sickle cell patients with the units of red blood cell transfusions

*Variables*	Cases with RBC* transfusions of less than 10 units	*p-value*	*Cases with RBC transfusions of 10 units or higher*	*p-value*	*Cases with RBC transfusions of 50 units or higher*	*p-value†*
Prevalence	32.7% (88)	ns‡	38.2% (103)	ns	28.9% (78)	ns
Associated thalassemias	45.4% (40)	ns	43.6% (45)	ns	42.3% (33)	ns
Mean RBC units	*4.0 ± 2.6* *(0-9) *	*0.000*	*21.2 ± 10.0* *(10-47)*	*0.000*	*97.0 ± 52.4* *(50-264)*	*0.000*
Mean age (year)	28.4 ± 10.1 (13-59)	ns	28.5 ± 8.5 (15-56)	ns	28.9 ± 8.9 (14-56)	ns
Male ratio	*37.5% (33)*	*<0.05*	*48.5% (50)*	*<0.01*	*62.8% (49)*	*<0.001*

*Red blood cell †Difference between the first and third groups ‡Nonsignificant (*p*>0.05

**Table-II:** Sickle cell patients with associated disorders

*Variables*	Cases with RBC* transfusions of less than 10 units	*p-value*	*Cases with RBC transfusions of 10 units or higher*	*p-value*	*Cases with RBC transfusions of 50 units or higher*	*p-value†*
Painful crises per year	*2.0 ± 3.8 (0-24)*	*0.000*	*4.5 ± 6.5 (0-36)*	*0.031*	*7.2 ± 8.6 (0-36)*	*0.000*
Digital clubbing	*3.4% (3)*	ns	*2.9% (3)*	*<0.05*	*7.6% (6)*	*<0.05*
COPD§	*1.1% (1)*	ns	*0.9% (1)*	*<0.001*	*7.6% (6)*	*<0.001*
Leg ulcers	*6.8% (6)*	*<0.05*	*12.6% (13)*	ns	*19.2% (15)*	*<0.001*
Stroke	*3.4% (3)*	ns	*2.9% (3)*	*<0.001*	*11.5% (9)*	*<0.001*
CRD¶	*1.1% (1)*	*<0.001*	*8.7% (9)*	ns	*14.1% (11)*	*<0.001*
Pulmonary hypertension	*5.6% (5)*	*<0.001*	*14.5% (15)*	ns	8.9% (7)	ns
Cirrhosis	3.4% (3)	ns	3.8% (4)	ns	2.5% (2)	ns
CHD¶	9.0% (8)	ns	7.7% (8)	ns	8.9% (7)	ns
Exitus	3.4% (3)	ns	5.8% (6)	ns	2.5% (2)	ns

*Red blood cell †Difference between the first and third groups ‡Nonsignificant (*p*>0.05)

§Chronic obstructive pulmonary disease ¶Chronic renal disease **Coronary heart disease

There were 15 cases without any RBC transfusion in their lives with a mean age of 26.9 ± 6.7 (17-39) years, and the prevalences were 7.2% and 3.7% in females and males, respectively (*p*<0.05). The mean age of cases with RBC transfusion or transfusions in their lives was 28.7 ± 9.2 (13-59), and the difference between the cases with and without RBC transfusion or transfusions was nonsignificant (*p*>0.05). There were 27 cases (10.0%) without any painful crisis in their lives with a mean age of 35.9 ± 9.6 (18-58) years, and the prevalences were 13.8% and 6.0% in females and males, respectively (*p*<0.001). Interestingly, the mean age of cases with painful crises in their lives was 27.7 ± 8.7 (13-59), and it was significantly lower than the cases without any crisis in their lives (*p*<0.000). There was a progressive and significant increase according to the male ratio from the first towards the third groups (37.5%, 48.5%, 62.8%, *p*<0.05 between all).

There was not any patient with regular alcohol consumption among the study cases. Although the prevalences of cirrhosis, CHD, and exitus were similar in the three groups (*p*>0.05 between all), there were progressive and significant increases according to mean painful crises per year, digital clubbing, COPD, leg ulcers, stroke, CRD, and pulmonary hypertension from the first towards the third groups (*p*<0.05, nearly in all steps) (Table-II). Mean ages of the mortal cases were 29.1 ± 9.9 (19-45) and 26.2 ± 6.6 (19-39) years in females and males, respectively (*p*>0.05). On the other hand, five of the CRD cases were on hemodialysis, and one with renal transplantation. Although antiHCV was positive in two of the cirrhotics, HCV RNA was detected as negative by polymerase chain reaction in both.

## DISCUSSION

Painful crises are nearly pathognomonic for the SCDs. For example, only 10.0% of the study cases had no crisis in their lives in the present study. The mean age of cases with crises was significantly lower than the cases without (27.7 versus 35.9 years, *p*<0.000). So frequency of painful crises indicates clinical severity of the SCDs, and the prevalences of cases without any crisis in their lives were significantly lower in males (13.8% versus 6.0%, *p*<0.001).

Infections may be the most common precipitating factors of the crises, probably by increasing leukocyte and thrombocyte numbers in circulation, and eventually enhancing inflammatory process of the endothelium. SCDs cases were immunocompromised due to a variety of reasons including chronic endothelial damage induced end-organ insufficiencies, the permanent inflammatory process all over the body, and frequent hospitalizations. Due to the repeated infarctions, an asplenism develops in early years of life. Terminal consequence of asplenism is an increased risk of infections, particularly with encapsulated bacteria. The causes of death were infection in 56% of infants in a previous study.^12^

In another study, the peak incidence of death occured between one and three years of age in children, and the deaths were predominantly due to pneumococcal sepsis in patients younger than 20 years of age.^13^ Therefore hydroxyurea treatment should be initiated in infancy to prevent chronic endothelial damage induced end-organ insufficiencies including autosplenectomy, to relieve permanent inflammatory process all over the body, to decrease frequency of hospitalizations, and eventually to restore immunity against infectious agents.^14^

Multiorgan failures, developed on the chronic background of SCDs, are not unusual during acute painful crises. During severe crises, RBC transfusions can provide adequate tissue oxygenation and immunity, and prevent intractable pain, dissemination of infections, and end-organ failures. Due to the severity of pain, narcotic analgesics are usually required to control them^15^, but according to our practice, simple RBC transfusions are highly effective during severe crises, both to relieve pain and to prevent sudden deaths secondary to multiorgan failures. Although narcotic analgesics can relieve pain alone, they can not reverse the underlying destructive process. So frequency of RBC transfusions may be another indicator of clinical severity, and the prevalences of cases without any RBC transfusion in their lives were also lower in males (7.2% and 3.7%, *p*<0.05). According to our practice, simple RBC transfusions should be preferred instead of RBC exchange due to possibility of less units of RBC requirements, to decrease potential risks of hypertransfusion including alloimmunization, infections, and iron overload, absence of the need of an expert, the lower cost of procedure, and the shorter time to find the less units of RBC. The shorter time to find the less units of RBC for simple transfusions may particularly be life saving in such mortal patients.

Severe pain may be a result of tissue hypoxia secondary to the chronic endothelial inflammation caused by the interactions between rigid cells, endothelial cells, leukocytes, and platelets. The adverse actions of platelets and neutrophils on endothelium may particularly be important. For example, leukocytosis even during silent periods was an independent predictor of severity of the SCDs^12^, and it was associated with increased risk of stroke.^16^ On the other hand, leukocytosis and thrombocytosis are acute phase reactants that are nearly present in all SCDs patients even during the silent periods, and they indicate presence of a permanent inflammatory process all over the body. The continuous inflammatory process alone causes additional atherosclerotic process and weight loss in the SCDs cases.^17^ So the mean weight and body mass index (BMI) were significantly retarded in the SCDs cases.^17^ Probably due to the significantly lower mean body weight and BMI, mean values of the low density lipoprotein cholesterol, alanine aminotransferase, and systolic and diastolic BPs were also lower in such cases^17^, which can be explained by definition of the metabolic syndrome.^18^^,^^19^

The rigid cells induced chronic endothelial damage causes tissue hypoxia, infarction, and end-organ insufficiencies even in the absence of obvious vascular occlusions on the chronic background of damaged and edematous endothelium all over the body. Even there were patients with severe vision and hearing losses among our study cases. The digital clubbing and recurrent leg ulcers may also indicate chronic tissue hypoxia in such patients. The chronic endothelial inflammation, initiating at birth, eventually terminates with a shortened survival in such patients. For example, the mean survivals were 42 and 48 years in the literature^20^, whereas the mean ages of mortal cases were 26.2 and 29.1 years in males and females in the present study, respectively. The great differences may be due to the initiation of hydroxyurea treatment in infancy in developed countries.^14^ Although the lower mean age of mortality in males, the difference was nonsignificant probably due to the small number of cases in the present study (*p*>0.05). Similarly, females have a longer overall survival all over the world.^21^

In conclusion, the significantly higher painful crises per year, digital clubbing, COPD, leg ulcers, stroke, CRD, pulmonary hypertension, and male ratio of the third group, the lower male ratio of patients without any RBC transfusion in their lives, the lower male ratio of patients without any painful crisis in their lives, the lower mean ages of the male SCDs patients with mortality, and the longer overall survival of females in the world could not be explained by well known strong atherosclerotic effects of smoking alone. Instead it may be explained by the dominant role of male sex in life.
